# Characterization of heme binding to recombinant α_1_-microglobulin

**DOI:** 10.3389/fphys.2014.00465

**Published:** 2014-12-04

**Authors:** Elena Karnaukhova, Sigurbjörg Rutardottir, Mohsen Rajabi, Lena Wester Rosenlöf, Abdu I. Alayash, Bo Åkerström

**Affiliations:** ^1^Laboratory of Biochemistry and Vascular Biology, Division of Hematology Research and Review, Center for Biologics Evaluation and Research, Food and Drug AdministrationSilver Spring, MD, USA; ^2^Division of Infection Medicine, Department of Clinical Sciences in Lund, Lund UniversityLund, Sweden; ^3^Division of Therapeutic Proteins, Office of Biotechnology Products, Office of Pharmaceutical Science, Center for Drug Evaluation and Research, Food and Drug AdministrationSilver Spring, MD, USA

**Keywords:** alpha-1-microglobulin, heme binding, surface plasmon resonance, circular dichroism

## Abstract

**Background**: Alpha-1-microglobulin (A1M), a small lipocalin protein found in plasma and tissues, has been identified as a heme^1^ and radical scavenger that may participate in the mitigation of toxicities caused by degradation of hemoglobin. The objective of this work was to investigate heme interactions with A1M *in vitro* using various analytical techniques and to optimize analytical methodology suitable for rapid evaluation of the ligand binding properties of recombinant A1M versions.

**Methods**: To examine heme binding properties of A1M we utilized UV/Vis absorption spectroscopy, visible circular dichroism (CD), catalase-like activity, migration shift electrophoresis, and surface plasmon resonance (SPR), which was specifically developed for the assessment of His-tagged A1M.

**Results**: The results of this study confirm that A1M is a heme binding protein that can accommodate heme at more than one binding site and/or in coordination with different amino acid residues depending upon heme concentration and ligand-to-protein molar ratio. UV/Vis titration of A1M with heme revealed an unusually large bathochromic shift, up to 38 nm, observed for heme binding to a primary binding site. UV/Vis spectroscopy, visible CD and catalase-like activity suggested that heme is accommodated inside His-tagged (tgA1M) and tagless A1M (ntA1M) in a rather similar fashion although the His-tag is very likely involved into coordination with iron of the heme molecule. SPR data indicated kinetic rate constants and equilibrium binding constants with K_D_ values in a μM range.

**Conclusions**: This study provided experimental evidence of the A1M heme binding properties by aid of different techniques and suggested an analytical methodology for a rapid evaluation of ligand-binding properties of recombinant A1M versions, also suitable for other His-tagged proteins.

## Introduction

Heme (iron-protoporphyrin IX) is a prosthetic group of hemoglobin (Hb), myoglobin and many other hemoproteins that perform a wide range of biological functions such as oxygen delivery, iron storage, electron transfer, and substrate oxidation (Smith et al., 2010). However, once released from Hb and other hemoproteins, heme is capable of generating reactive oxygen species that can oxidatively damage lipids and proteins, leading to oxidative stress and cell and tissue damage (Jeney et al., 2002; Buehler et al., 2011). Heme toxicity is seen in several pathological conditions including hemolytic disorders such as malaria, β-thalassemia and sickle-cell disease (Vinchi et al., 2013; Dutra and Bozza, 2014). Recently, heme was shown to bind specifically to the Toll-like Receptor (TLR4) leading to endothelial cell activation and vaso-occlusion in murine sickle cell disease (Belcher et al., 2014).

Hb, the most abundant heme-containing protein in the circulation, is the major source of free heme due to catabolism of extracellular Hb in hemolytic disorders. Exogenous sources of free heme include red blood cell (RBC) preparations during storage (Buehler et al., 2011), heme-based therapeutics (such as Panhematin, hemin for injection, and heme arginate) that are used for the treatment of porphyrias (Tenhunen and Mustajoki, 1998; Siegert and Holt, 2008), and Hb-based blood substitutes (Alayash, 2014). In the circulation, the free heme redox toxicity is mitigated by several innate defense mechanisms, including heme sequestration by plasmatic major heme scavengers hemopexin, albumin, and some other proteins (Ascenzi et al., 2005; Buehler et al., 2011). A1M, also known as protein HC, has emerged among anti-oxidative defense mechanisms as a tissue housekeeping protein which is capable of capturing heme and free radicals (Allhorn et al., 2002; Åkerström et al., 2007; Olsson et al., 2012; Åkerström and Gram, 2014), as well as reducing oxidative lesions (Allhorn et al., 2005; Olsson et al., 2008; Rutardottir et al., 2013). This small heterogeneously charged glycoprotein (~26 kDa) belongs to the lipocalin protein family, a group of small extracellular proteins that are known for a great diversity of their primary sequence, but share a well-conserved tertiary structure (Åkerström et al., 2000; Flower, 2000). The lipocalins share a common fold, a β-barrel consisting of eight antiparallel β-strands with a closed bottom, an open end and a hydrophobic pocket which can carry small lipophilic ligands (Ganfornina et al., 2006). Whereas precise physiological functions of A1M and many other lipocalins are not defined yet, these proteins are known for their ligand-binding properties and thus, seem to play an important role in binding, storage and transport of small lipophilic molecules (Breustedt et al., 2006). A1M was shown to bind heme in different species (Allhorn et al., 2002; Larsson et al., 2004). Because of its radical and heme binding properties and antioxidant potential, A1M is being considered for several therapeutic applications (Åkerström and Gram, 2014), and recombinant His-tagged A1M has been successfully employed for *in vivo* treatment of preeclampsia in a sheep model (Wester-Rosenlöf et al., 2014) and Hb-induced glomerular dysfunction in rats (Sverrisson et al., 2014).

A previous study suggested that each A1M-molecule binds two heme-groups (Siebel et al., 2012). The aim of this study was to characterize the interactions of recombinant A1M with heme using various analytical techniques. Our analytical methodology included (a) UV/Vis monitoring of the A1M titration by heme, (b) evaluation of a possible involvement of the recombinant A1M His-tag into a coordination with iron of the heme molecule, (c) visible circular dichroism (CD) to characterize heme/A1M interactions by aid of protein-induced chirality (induced CD), and (d) development of surface plasmon resonance (SPR) analysis suitable for the immobilization of recombinant A1M by the His-tag *per se* to study the interactions with heme and/or other ligands without removal of the tag.

## Materials and methods

### Materials

Hemin, dimethyl sulfoxide (DMSO), L-His, imidazole, and essentially fatty acid free human serum albumin (HA) were purchased from Sigma Chemical Co. (St. Louis, MO). Recombinant human A1M with an N-terminal His8-tag (tgA1M) was expressed and purified in *E.coli* as described (Kwasek et al., 2007), with the addition of ion-exchange chromatography and size exclusion purification steps performed as follows. The protein solution was applied to a column of DEAE-Sephadex A-50 (GE Healthcare, Uppsala, Sweden) equilibrated with the starting buffer (20 mM Tris-HCl, pH 8.0). A1M was eluted at a flow rate of 1 ml/min using a linear pH gradient consisting of 250 ml starting buffer and 250 ml elution buffer (20 mM Tris-HCl, 0.15 M NaCl, pH 8.0). Size-exclusion chromatography was run on a Superose 12 column obtained from GE Healthcare using Äkta purifier 10 system (GE Healtcare) run at a flow-rate of 1 ml/min. Notag A1M (ntA1M) was a obtained from A1M Pharma (Lund, Sweden). Research grade CM5 sensor chips, NTA sensor chip, 1-ethyl-3-(3-dimethyl-aminopropyl)-carbodiimide hydrochloride (EDC), N-hydroxysuccinimide, ethanolamine–HCl, HBS–P buffer (0.01 M HEPES, pH 7.4, 0.15 M NaCl, 0.005% surfactant P20), Phosphate buffer saline, 10X (PBS) (0.1 M phosphate buffer with 27 mM KCl and 1.37 M NaCl, pH 7.4), acetate buffer (pH 5.0), NaOH (50 mM), and deionized water were from GE Healthcare (Piscataway, NJ). Anti-His mouse IgG1 monoclonal antibodies were purchased from R&D Systems (Minneapolis, MN). Hemopexin (HPX) was from Athens Research Technologies (Athens, GA). Other chemicals were analytical grade from Fisher Scientific (Fair Lawn, NJ).

### Preparation of protein solutions

Protein stock solutions (22 and 45 μM) of A1M, HA, HPX were prepared in 20 mM Tris-HCl, 0.15 M NaCl, pH 8.0 (Tris buffer), aliquoted and stored frozen at −70°C until use. A1M samples in PBS or Tris buffer were prepared by dilution of the concentrated stock solutions prior to use. Concentrations of the reference proteins HA and HPX were determined spectrophotometrically using a molar extinction coefficient of 5.5 × 10^4^ M^−1^cm^−1^ for HA (Gill and von Hippel, 1989) and 1.1 × 10^5^ M^−1^cm^−1^ for apo-HPX (Eskew et al., 1999).

### Preparation of imidazole, L-His and His8 solutions

Imidazole and L-His solutions were prepared in Tris buffer at concentrations 100 mM and 50 mM, respectively, to provide a large excess over the heme to be added. A His-octapeptide was synthesized at CBER Core facility (Silver Spring, MD) and dissolved to 100 mM in Tris buffer prior to adding of heme aliquots followed by UV/Vis spectral measurements.

### Preparation of heme solution

Heme stock solutions (2.4 and 1.2 mM) were freshly prepared in DMSO for each experiment, purged with argon and stored (protected from light) at 4°C during use. The heme concentrations in the stock solutions were determined spectrophotometrically using heme molar extinction coefficient of 170,000 M^−1^ cm^−1^ at λ_max_ 404 nm (Collier et al., 1979).

### UV/Vis measurements

UV/Vis electronic absorption spectra were measured on Agilent HP8453 UV-visible spectrophotometer (Agilent Technologies Deutschland GmbH, Germany) at 25 ± 0.2°C in a quartz cuvette of 1 cm pathlength. The measurements were performed in the range of 250–700 nm for A1M interactions with heme. UV/Vis differential spectra of the heme/A1M samples were measured using A1M as a blank.

### A1M titration with heme

Titration of A1M samples with heme was performed using 45 μM A1M preparations in Tris buffer. Heme concentration was increased stepwise by addition of 2 μL aliquots of 2.1 mM heme stock solution in DMSO to a 1 mL of 45 μM protein solution. UV/Vis and CD recordings were repeated until no significant alterations were observed, before the next heme aliquot was added. During titration the samples were maintained at room temperature, protected from light and periodically flashed by argon. Initial 45 μM A1M solutions served as a blank. The total amount of DMSO in the titration samples did not exceed 2% v/v. Titration of A1M with heme was carried out until the ligand-to-protein molar ratio (L/P) was 1.4. Since signs of turbidity and slight precipitation were observed for the samples with high heme content, only the data up to L/P 1.0 were included.

### Migration shift electrophoresis

Tris buffer with 5 μM tgA1M or ntA1M was incubated for 15 min at room temperature with 50, 5, 0.5, and 0 μM heme. Samples were mixed with equal amounts of sample buffer for native PAGE, pH 6.8, and subjected to electrophoresis on 12% Criterion™ TGX™ Precast stainfree gels (Bio-Rad) under non-denaturing and non-reducing conditions. The gel was first imaged by tryptophan fluorescence with a BioRad GelDoc MP Imaging system and then stained with Coomassie Brilliant Blue R-250 (BDH Chemicals, Ltd. Poole, UK).

### CD measurements

CD measurements were performed using a Jasco J-815 Spectropolarimeter (JASCO Co., Japan) with the temperature maintained at 25 ± 0.2°C by a Peltier thermostat. The spectra were measured at a scan speed of 100 nm/min, bandwidth of 1.0 nm, and resolution of 0.2 nm and accumulated in triplicate. All samples containing heme were measured for protein-induced CD in the near-UV—visible range from 700 to 300 nm (referred to as visible CD). To accumulate the induced CD, A1M preparations were used at a concentration ~45 μM in Tris buffer, and the measurements of heme/A1M samples were performed in a quartz cuvette with 1 cm pathlength. Protein alone was used as a blank. Heme/HPX and heme/HA samples were recorded at the same concentration, but in a cuvette with a shorter pathlength (5 mm) because of the strong induced CD, and then the spectra were normalized. An ellipticity of CD spectra was expressed in millidegrees (mdeg).

### Surface plasmon resonance

SPR experiments were performed using Biacore T200 (GE Healthcare, Piscataway, NJ). About 18,000 response units (RU) anti-His mouse IgG1 monoclonal antibody (R&D Systems, Minneapolis, MN) were immobilized on CM5 sensors using the amine coupling kit. The tagged protein was captured by injecting tgA1M (10 μL/min) for 360 s. Heme samples were prepared prior to injection by serial double dilutions of the initial 100 μM heme solution in PBS-P/5% DMSO to create a range over eight concentrations (from 100 to 0.625 μM). Heme samples prepared at pH 7.2 and pH 8 were injected over captured A1M for 2 min with a flow 30 μL/min at 25°C. The dissociation was monitored for 10 min. Data were analyzed using the Biacore T200 evaluation software (GE Healthcare), subtracting the reference surface (immobilized anti-His mouse IgG1 antibody from the captured A1M surface) and buffer control signals from each curve. Data were globally fitted by simultaneous numerical integration to the association and dissociation parts of the interaction, using the heterogeneous ligand kinetic analysis models. Steady-state equilibrium was reached during the association phase, global fitting produced reproducible kinetic parameters. Experimental *R*_maximum response_ (*R*_max_) values obtained by global fitting were comparable to theoretical *R*_max_ values calculated from the molecular masses of the interacting proteins and the immobilization level. For the interactions with an equilibrium achieved, *K*_D_ was also calculated by an equilibrium analysis.

The T200 BIAevaluation software (version 1.0; Biacore AB, Uppsala, Sweden) offers various reaction models to perform complete kinetic analyses of the sensorgrams, from which the heterogeneous ligand binding model were applied. This model describes an interaction between one analyte and two independent ligands or two binding sites of one ligand. The binding curve obtained is simply the sum of the two independent reactions.

### Catalase-like activity assay

Stability of the Soret band intensity and heme accessibility to hydrogen peroxide in heme/tgA1M and heme/ntA1M samples in comparison with heme alone in Tris buffer were evaluated using the catalase-like activity assay, as described earlier (Karnaukhova et al., 2012) with minor modifications. Briefly, heme/A1M equimolar (L/P 1.0) samples were prepared by adding 21 μL of 2.1 mM heme stock solution in DMSO to 1 mL of 45 μM solutions of tgA1M and ntA1M in Tris buffer. The samples were incubated at room temperature for 20 h, protected from light. The complex formation was confirmed by a stable Soret band in the UV/Vis spectra, i.e., λ_max_ at 411 nm typical for heme/tgA1M (L/P 1.0) and λ_max_ at 389 nm typical for heme/ntA1M (L/P 1.0), respectively. As a reference, a heme sample in Tris buffer was freshly prepared the same way and used immediately in view of its limited stability in aqueous solutions. The samples were normalized to adjust their Soret bands to approximately the same intensity (~0.56 AU) by dilution with Tris buffer. After addition of a 7 μL aliquot of 50 mM H_2_O_2_ solution to 1 mL of the sample (~8-fold excess over the heme complex content), the absorption spectra were taken at the following time points: 30 s, 1, 2, 4, 6, 8, and 10 min.

## Results and discussion

### UV/Vis assessment

**Figure 1:**
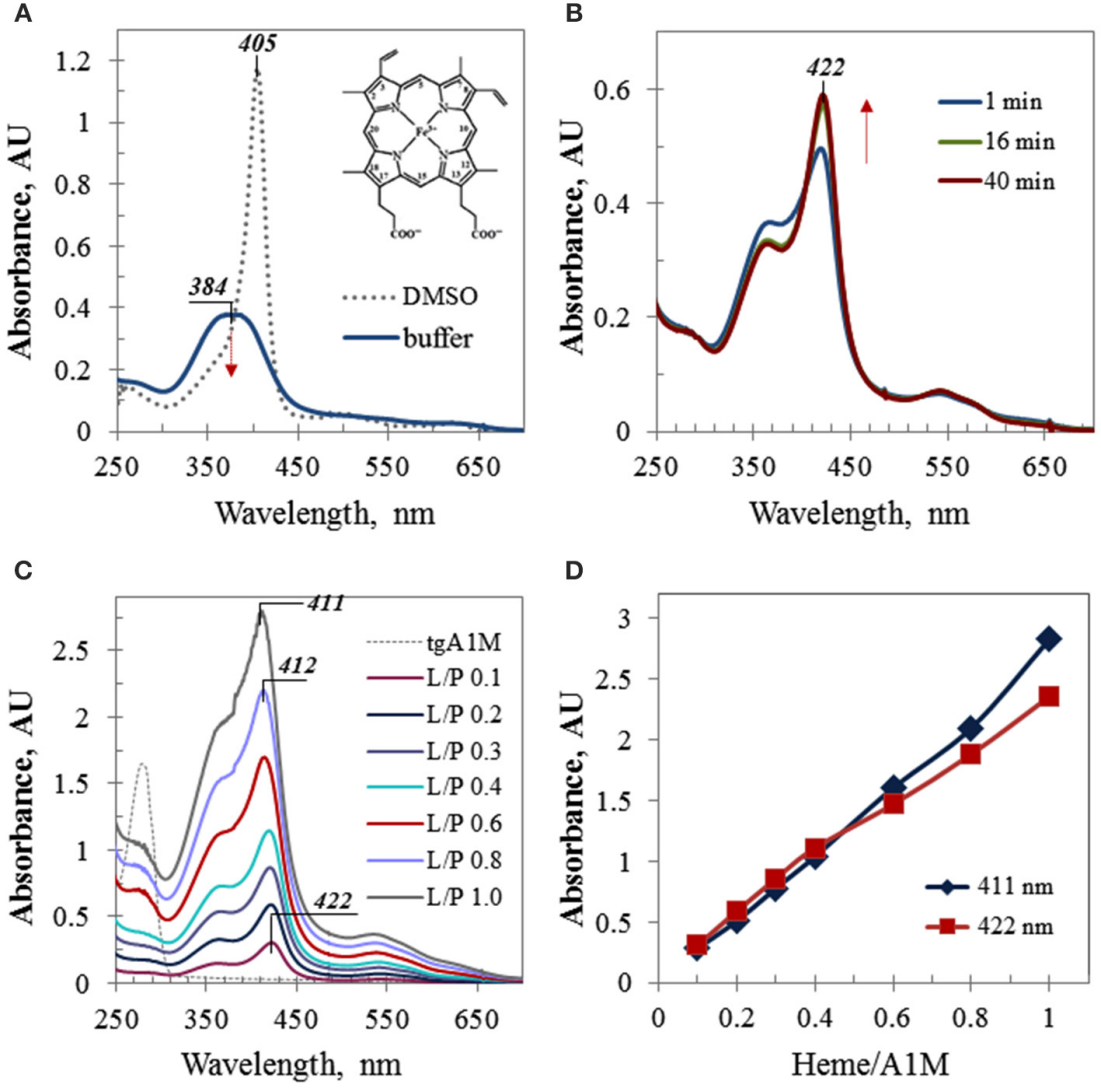
**UV/Vis electronic absorbance data. (A)** UV/Vis spectra of heme in Tris buffer and DMSO; the same aliquot of 1.28 mM heme stock solution was added to 1 mL of buffer or DMSO, respectively; inset shows heme structure; **(B)** time-course absorbance spectra of 45 μM A1M sample containing 0.1 molar equivalent of heme (L/P 0.1); **(C)** differential UV/Vis spectra of the A1M titration by heme (the initial spectrum of 45 μM A1M, shown by thin dotted trace, has been subtracted by running the A1M sample as a blank prior to adding heme); the spectra are shown for the time point 40 min; all titration samples were measured in 10 mm pathlength cuvette, except the last one (L/P 1.0) which spectrum has been recorded in 5 mm pathlength cuvette and then normalized to 10 mm path; **(D)** the absorbance intensities of the Soret band of heme complexes with A1M as plotted at the wavelengths 422 nm (λ_max_ at low L/P) and 411 nm (λ_max_ at higher L/P).

Due to its hydrophobic nature, free heme possesses a low solubility in aqueous media and has a tendency to aggregate. In organic solvents, such as ethanol or DMSO, heme has much better solubility and exhibits a strong Soret band at 404–405 nm, stable for few days. When an aliquot of a heme stock solution in DMSO was added to a buffer solution, a broad Soret band around 380 nm with a relatively low intensity was observed which further decreased with time (Figure 1A). However, when a heme aliquot was added to the same buffer (Tris, pH 8), containing A1M, the observed spectral alterations included (a) a drastically red-shifted Soret band (~38 nm shifted in comparison with the spectrum of heme alone), (b) increasing intensity of this band, which remained stable when reaches maximum, and (c) characteristic isosbestic point for the spectra taken at different time-points (Figure 1B). These features are indicative of a complex formation between heme and A1M. Figure 1C shows UV/Vis absorption spectra of the A1M titration with heme which was performed by adding of small calculated aliquots of heme stock solution in DMSO to 45 μM A1M in Tris buffer to increase the heme content in the heme/A1M mixture by 0.1 molar equivalent steps. As heme accomodation in A1M takes time (Figure 1B), each spectrum shown in Figure 1C was taken at a time point not less than 40 min after adding next heme aliquot to allow the reaction mixture to reach its equilibrium state, when no further significant alterations in the intensity or position of the Soret band were observed. Further titration resulted in a gradual increase of the Soret band intensity that was almost linearly proportional to the heme content in the sample (Figures 1C,D), and in the characteristic alteration of the Soret band λ_max_ from 422 nm (at low L/P of 0.1 and 0.2) to λ_max_ at 411–412 nm observed for higher L/P.

As evident from the spectra obtained for the samples with heme content corresponding to low ligand-to-protein molar ratios (L/P) such as 0.1 and 0.2, heme in A1M solution exhibited a Soret band at 422 nm that is strongly red-shifted in comparison with heme in buffer alone (384 nm, Figure 1A). A bathochromic shift in UV/Vis absorption spectra provides evidence of a ligand complex formation with the host protein (Zsila et al., 2003, 2005; Neya et al., 2010). A large excess of the protein over heme promotes binding of heme primarily to the protein site(s) of high affinity, and a heme co-ordination by amino acid residues inside the primary binding site(s) may result in a higher energy level and thus, a red-shifted Soret band. Interestingly, the bathochromic shift observed for heme/A1M (~38 nm) is much larger than those of plasma major heme scavengers hemopexin and human albumin which possess high affinities for heme and well-fit heme primary binding sites, and are characterized by Soret bands at 413 and 405 nm, respectively.

A recent report (Siebel et al., 2012) suggested binding of two molecules of heme to each A1M molecule. This is supported by the results shown in Figure 1. Titration of A1M with heme (Figures 1C,D) suggested heme binding to a primary binding site of high affinity at low L/P and also to a hypothetical secondary site of lower affinity at higher L/P. With increasing heme content, heme continued to fill both binding sites, and at higher L/P values (starting L/P 0.6) the intensity of the Soret band at 411 nm prevailed over that at 422 nm (Figure 1D). At a heme-to-protein ratio higher than 1.2 (i.e., heme concentrations beyond 50 μM), some turbidity and slight precipitation could be observed. UV/Vis spectra of the supernatant showed a broad band around 405 nm with a shoulder at 355 nm, very likely due to an overlapping of the Soret bands corresponding to aforementioned sites, free and aggregated heme in the solution, and non-specific heme binding at the protein surface, which, however, were beyond the scope of this study.

Thus, UV/Vis titration provided strong experimental evidence of the heme binding to A1M. The results were compatible with two heme binding sites as suggested previously (Siebel et al., 2012) and also raised the question of the nature of this extremely red-shifted Soret band.

**Figure 2:**
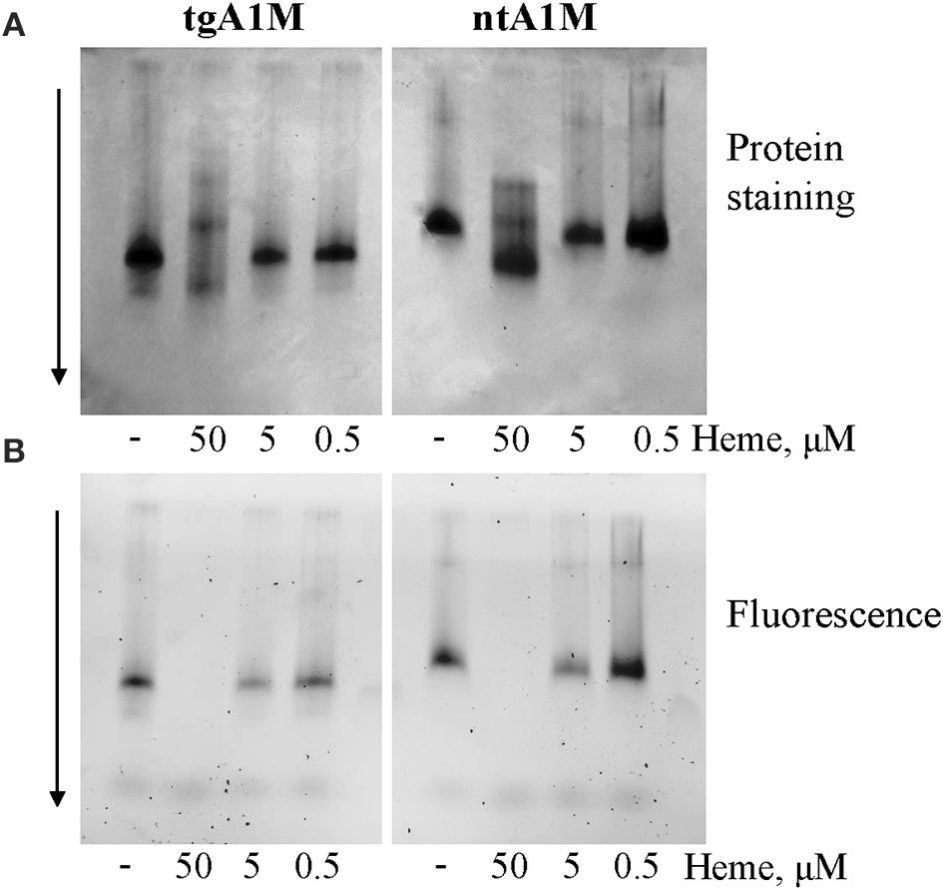
**Native gel data for heme/A1M samples.** tgA1M and ntA1M (5 μM) were incubated with 50, 5, and 0.5 and 0 μM heme for 15 min at room temperature, and separated by electrophoresis in a 12% Criterion™ TGX™ Precast stainfree Gels under non-denaturing and non-reducing conditions. The gels were either stained with Coommassie Brilliant Blue **(A)** or imaged by tryptophan fluorescence with a digital BioRad imager **(B)**.

It is well known that heme can interact with His-tags (Asher and Bren, 2010) and can be involved in *bis*-His (hexa-) coordination (Owens et al., 2013). Therefore, the large bathochromic shift observed for heme/A1M samples (Figure 1) posed the question whether the His-tag may have an impact on the heme-binding. First, a native PAGE was run demonstrating that both His-tagged A1M (tgA1M) and tagless A1M (ntA1M) indeed bind the heme molecule (Figure 2). Thus, the migration of both tg- and nt-A1M was slowed by the heme bound (panel A), and the tryptophan-fluorescence completely quenched (panel B). A similar dose-response of migration shift and fluorescence quenching was noted for the two forms, suggesting similar affinities and coordination for tg- and nt-A1M. No effect on migration or fluorescence could be seen on the negative control ovalbumin by addition of heme.

**Figure 3:**
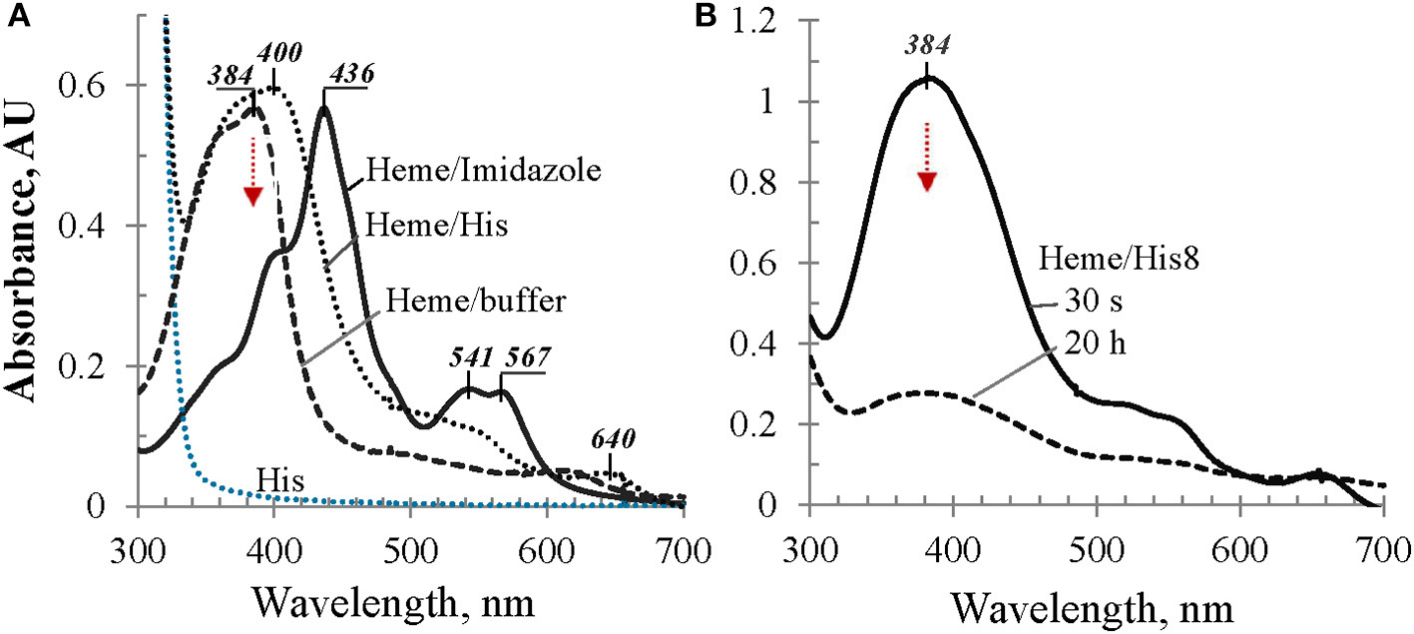
**UV/Vis spectral evaluation of possible bathochromic shift due to imidazole-containing compounds. (A)** Absorbance spectra of heme in Tris buffer alone, in 0.1 M L-His, and in 0.1 M imidazole solutions, as labeled; **(B)** heme in 50 mM solution of His8-peptide in Tris buffer: 30 s after adding heme aliquot and 20 h later, as marked; for details see Materials and Methods.

To evaluate a possible involvement of the 8-mer His-tag in the hexa-coordination of heme iron, we first studied heme behavior in the mixtures with imidazole and L-His in comparison with heme in buffer alone (Figure 3A). In the L-His solutions in Tris buffer, heme exhibits a strong broad Soret band around 400 nm (Figure 3A) which is ~16 nm red-shifted in comparison with heme in buffer alone. Even more remarkable, added to the imidazole solution in buffer, heme exhibits a strong Soret band at 436 nm with a shoulder at around 400 nm and two well-defined bands in the visible range, at 541 and 567 nm (Figure 3A). Both experiments suggest that imidazole side chains of the His-tagged protein may participate in the heme iron hexa-coordination by a sandwich structure when heme content is low in comparison with the protein (such as L/P 0.1 and 0.2 discussed above). However, when we examine His-octapeptide which was specifically synthesized to verify whether His-tag alone may provide heme hexa-coordination to cause a large bathochromic shift, such as ~38 nm, no significant difference was observed between the spectra of heme in His8-peptide solution and heme in buffer solution alone (Figure 3B). The λ_max_ (384 nm) of the absorption band of heme/His8 sample (Figure 3B) was very much similar to that of heme in buffer alone (Figure 3A) as well as the decrease of its intensity in time (broken trace corresponding to 20 h in Figure 3B). Thus, contrary to imidazole and histidine, no sign of a complex formation with heme was observed for synthetic His-octapeptide under experimental conditions used.

**Figure 4:**
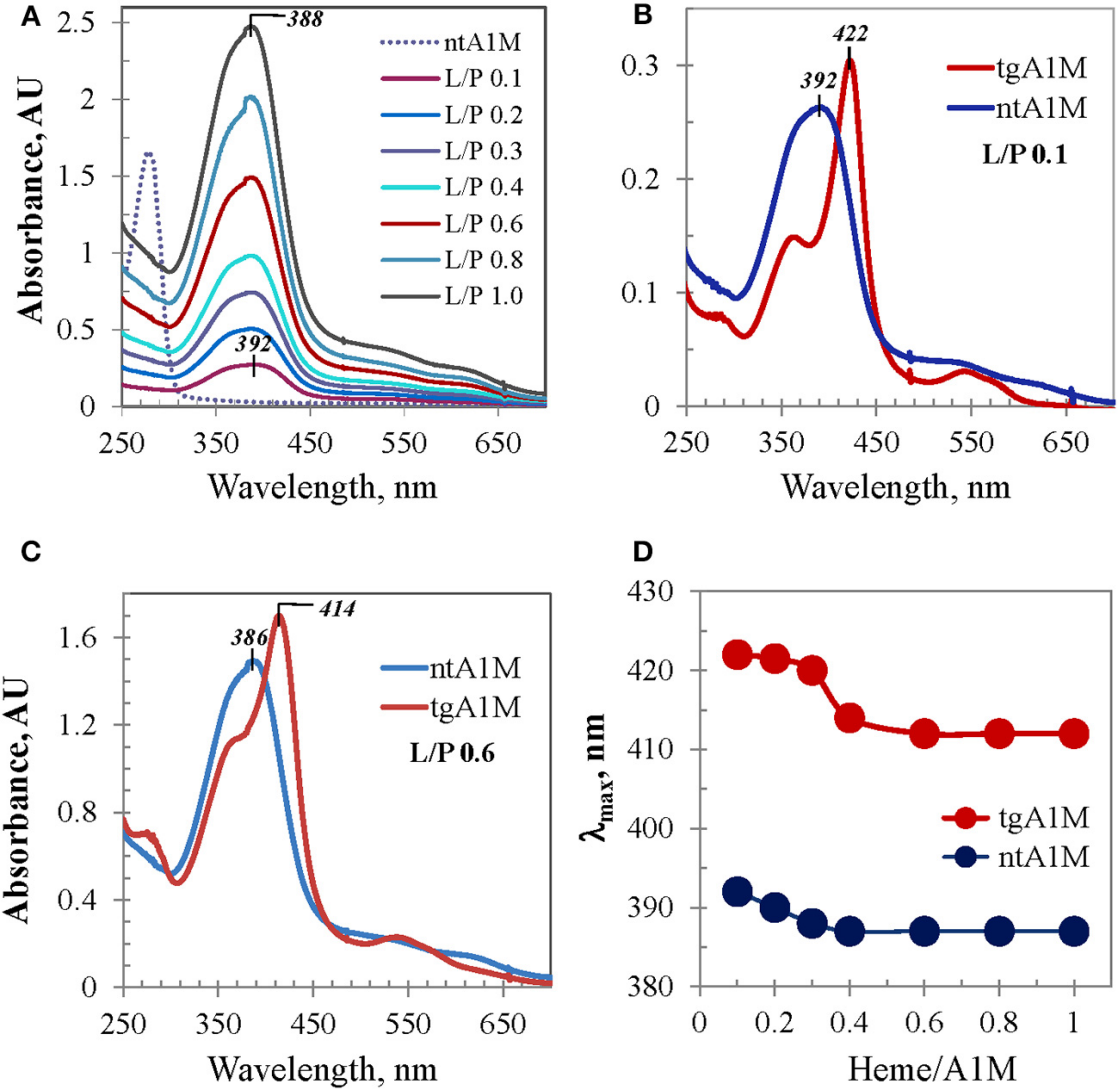
**UV/Vis absorbance data for ntA1M and comparison with tgA1M. (A)** UV/Vis titration of ntA1M by heme (under conditions used for tgA1M, Figure 1C); **(B,C)** UV/Vis spectra of heme complexes with tg- and nt-A1M samples at low (L/P 0.1) and higher (L/P 0.6) heme-to-A1M molar ratio; **(D)** alterations of the Soret band λ_max_ of heme/tgA1M and heme /ntA1M samples of the titration sets shown in Figures 1C, 3A.

This subject was further investigated with tagless A1M. Figure 4A shows differential UV/Vis titration spectra of ntA1M conducted the same way as that shown in Figure 1C. UV/Vis spectrum of the heme/ntA1M mixture of L/P 0.1 was characterized by a broad Soret band around 392 nm, which is only 8 nm red-shifted in comparison with that of heme in buffer alone (384 nm). UV/Vis spectra corresponding to further titration of ntA1M with heme demonstrated similar trends to the Soret band blue shifting, however from 392 to 388 nm (Figures 4A,D), and an almost linear dependence between the intensity of the Soret band and heme content in the sample (not shown) as those observed for tgA1M. Comparison of the two titration sets demonstrates a difference between each pair of the tgA1M and ntA1M samples of the same L/P at every step of the titration in terms of Soret band λ_max_ and shape of the spectra. As evident from the spectra overlays exemplified in Figure 4 for L/P 0.1 (panel B) and L/P 0.6 (panel C), UV/Vis spectra of heme/tgA1M samples are significantly more narrow than those of heme/ntA1M, and the λ_max_ values of the corresponding Soret bands differ by 30 nm and 28 nm, respectively. Figure 4D provides a summary of the λ_max_ values for the titration of tagged and tagless A1M variants over the entire heme concentrations range used in this study. This plot implicates a His-tag involvement in the heme interactions with A1M, but also demonstrates a characteristic two-phase titration profile suggesting that heme binding to both tgA1M and ntA1M proceeds at two (primary and secondary) binding sites.

### Induced circular dichroism

**Figure 5:**
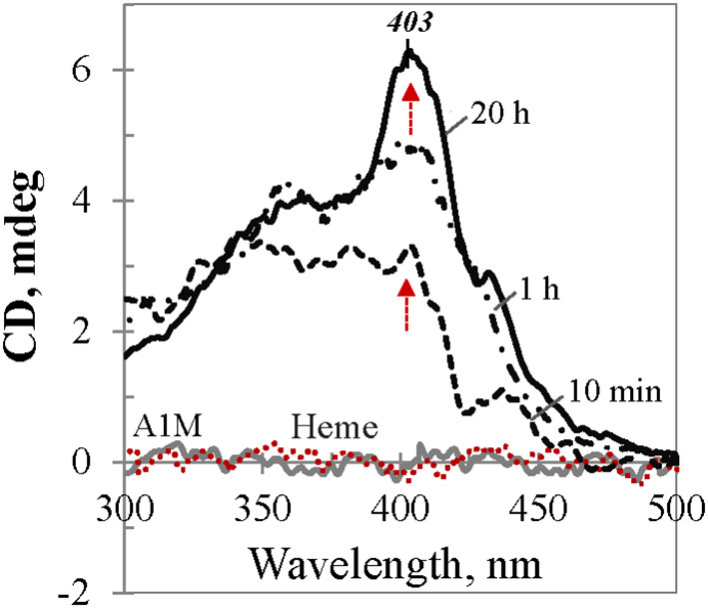
**CD dynamics of heme binding to tgA1M.** The spectra correspond to 40 μM heme in Tris buffer alone (red dotted trace at the baseline), and in 45 μM A1M solution at the time points 10 min, 1 h, and 20 h, respectively (black traces). Gray spectrum corresponds to 45 μM A1M in Tris buffer alone.

To provide more insight into the nature of the heme binding to A1M, we carried out a CD study in the visible range which may reflect heme induced optical activity due to its interaction with A1M, because otherwise heme *per se* is an optically inactive molecule. As demonstrated in Figure 5, neither heme nor A1M exhibited any CD in the visible range (dotted red and gray traces at zero-line, respectively). However, when the same amount of heme was added to the buffer solution containing A1M, a rapidly arising Cotton Effect (CE) was observed, a phenomenon known as protein-induced chirality (Zsila et al., 2004; Karnaukhova, 2007; Nagai et al., 2014). Further conformational changes (1 and 20 h traces are shown) were seen over time, with a slowly rising Soret CD band at 403 nm as the most prominent feature.

The induced CD spectra shown in Figure 5 for an equimolar mixture of heme and tgA1M, illustrate the dynamics of the complex formation. Contrary to UV/Vis absorption spectra, which reflect spectral properties of all heme species in the sample (free, bound to the protein internal binding site, or associated with protein at the surface), protein induced CD spectra, by definition, relate only to that part of heme which exhibits optical activity due to chiral perturbation to its structure per accomodation in the protein binding site. The CD spectra shown in Figure 5, in their original unsmoothed form, reflect ongoing conformational changes of the heme molecule. While at low heme content the binding to the A1M primary binding site proceeds relatively fast (with no further significant changes after ~40 min, not shown), heme binding to the secondary binding site proceeds slowly at higher heme-to-A1M ratio (Figure 5). It takes almost 20 h to achieve an equilibrium state with a well-defined positive CE at 403 nm and no further changes. The induced CE is in a good agreement with the λ_max_ of the Soret band in the complementary UV/Vis spectrum (which is ~411 nm for L/P 1.0).

**Figure 6:**
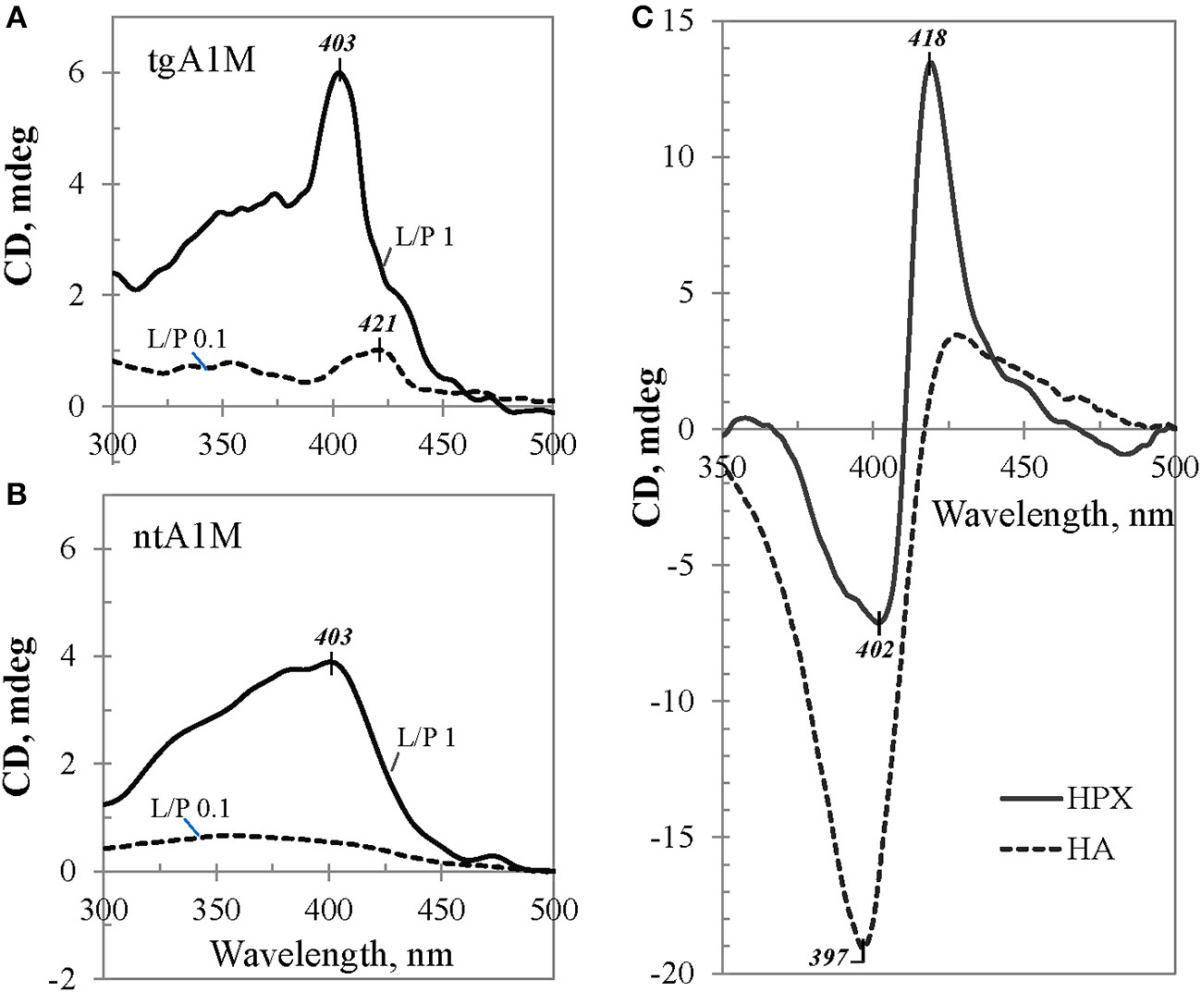
**Induced CD data.** Selected visible CD spectra of heme complexes with **(A)** tgA1M and **(B)** ntA1M, shown for L/P 0.1 (broken trace) and L/P 1.0 (solid trace), and **(C)** reference proteins HA and HPX. All samples were of approximately the same initial protein concentration, ~45 μM.

Figure 6 shows induced CD spectra of the heme complexes with tgA1M (panel A) and ntA1M (B) at L/P 0.1 and 1.0, after an equilibrium was achieved by each mixture (the spectra were measured at 20 h after adding heme). According to Figure 5A, the induced CD of the heme/tgA1M sample with L/P 0.1 demonstrates a small positive CE at 421 nm, thus reflecting the heme environment at the primary binding site. The CD spectrum corresponding to L/P 1.0 shows significantly different CE at 403 nm, very likely due to binding of the heme molecule at the secondary binding site, whereas a shoulder around 420 nm still suggest an overlap of the induced CD spectra from both binding sites. The induced CD spectra of heme/ntA1M samples (Figure 6B) are broader and of lower intensity of the induced CE. While CD spectrum corresponding to the sample with L/P 0.1 is weak, the induced CD spectrum of heme/ntA1M of L/P 1.0 shows CE at the same 403 nm as that observed for heme/tgA1M (panel A), although of lower intensity. Comparison of the induced CD data visualizes the impact of His-tag, but also indicates that ntA1M binds at least one of the heme-groups at the same binding site as the tagged A1M does, as evident from their CE at 403 nm.

The induced CE observed for heme/A1M samples were compared with those measured for heme/HPX and heme/HA samples of the same heme-to-protein molar ratio (Figure 6C). Although the three proteins are quite different, the intensity of the induced CD is generally determined by the strength of heme interactions with each protein and geometry of the binding site (Daviter et al., 2013). Therefore, higher intensities of the induced CD spectra in case of heme/HPX and heme/HA, in comparison with that of heme/A1M, suggest that heme binding sites in hemopexin and albumin are tighter and of higher affinity than those of A1M. Furthermore, while the CD titration spectra of heme/HPX and heme/HA at L/P 0.1 and up to 1.0 are indicative of one main heme binding site, the differences between heme/A1M CD spectra at low L/P and higher L/P support heme binding at two binding sites.

### SPR kinetics of heme binding to A1M

**Figure 7:**
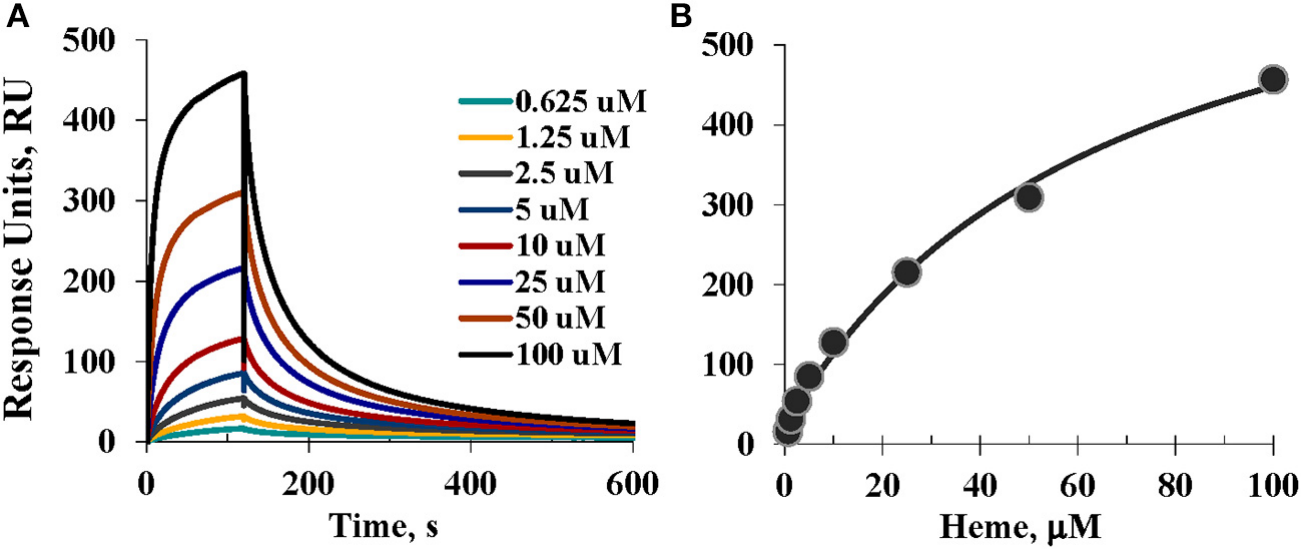
**SPR assessment. (A)** Sensorgrams of the heme binding to A1M captured by the anti-His mouse IgG1 monoclonal antibody immobilized on CM5 sensor chip; and **(B)** Steady-state equilibrium analysis and fitting; see details in Materials and Methods.

SPR (Biacore) was used to study the real-time kinetics of the heme interactions with A1M using different approaches. Our attempts to immobilize A1M directly on the CM5 surface were unsuccessful. Instead, we immobilized A1M by using the His-tag of the recombinant protein as an anchor for immobilization (Kimple et al., 2010). This approach also has the advantages of reducing the possible impact of the His-tag on coordination with heme, and allowing studies of other possible ligands besides heme. Therefore, we first explored immobilization of tgA1M by the His-tag using NTA chip as described elsewhere (Khan et al., 2006; Fischer et al., 2011). However, although tgA1M was successfully immobilized on the NTA sensor chip, we experienced continuous dissociation of the immobilized A1M from the surface, which was also reported for some other proteins (Willard and Siderovski, 2006; Clow et al., 2008). Instead, we used primary immobilization of anti-His mouse IgG1 monoclonal antibody (Franco et al., 2013). With this approach we were able to successfully capture the tgA1M to a highly pure homogeneous surface used for heme binding kinetics over a wide range of heme concentrations from 500 μM down to 0.625 μM. Interestingly, with a heme concentration range of 0.625–100 μM (Figure 7), the SPR data were a perfect fit to 1:1 Langmuir binding model, yielding kinetic rate constants k_a_ and k_d_ of 561.1 M^−1^s^−1^ and 7.75 × 10^−3^ s^−1^, respectively, and resulting in an equilibrium binding constant K_D_ of 13.82 μM. When the heme concentration range was extended to higher concentrations (up to 500 μM), the experimental data fitted better to a 1:2 binding model, thus supporting two binding site model suggested by Siebel and co-workers (Siebel et al., 2012). Using this model, the stronger affinity binding site was characterized by the association (k_a_) and dissociation (k_d_) rates 405.4 M^−1^s^−1^ and 2.85 × 10^−3^ s^−1^, respectively, thus resulting in a calculated K_D_ value of 7.02 μM. The binding site of lower affinity was characterized by k_a_ of 110.8 M^−1^s^−1^ and k_d_ of 0.02106 s^−1^, yielding a calculated K_D_ value of 190 μM. However, the accuracy of the K_D_ evaluation for the low affinity binding site can very likely be impacted by a heme tendency to aggregate at higher concentrations.

For the primary binding site, the SPR results reproducibly showed K_D_ values in the low micromolar range (as shown above, 13.82 and 7.02 μM) which is consistent with that determined earlier by a different technique (Larsson et al., 2004). It is noteworthy that at neutral buffer, such as pH 7.2 (data not shown) heme affinity to A1M was ~10-fold weaker than at pH 8, very likely due to a better heme solubility at more basic conditions. This observation seems to be in agreement with our CD data as a stronger intensity of the induced CD was observed for heme/A1M samples at pH 8 than at pH 7.2–7.4 (not shown).

### Catalase-like activity assessment

**Figure 8:**
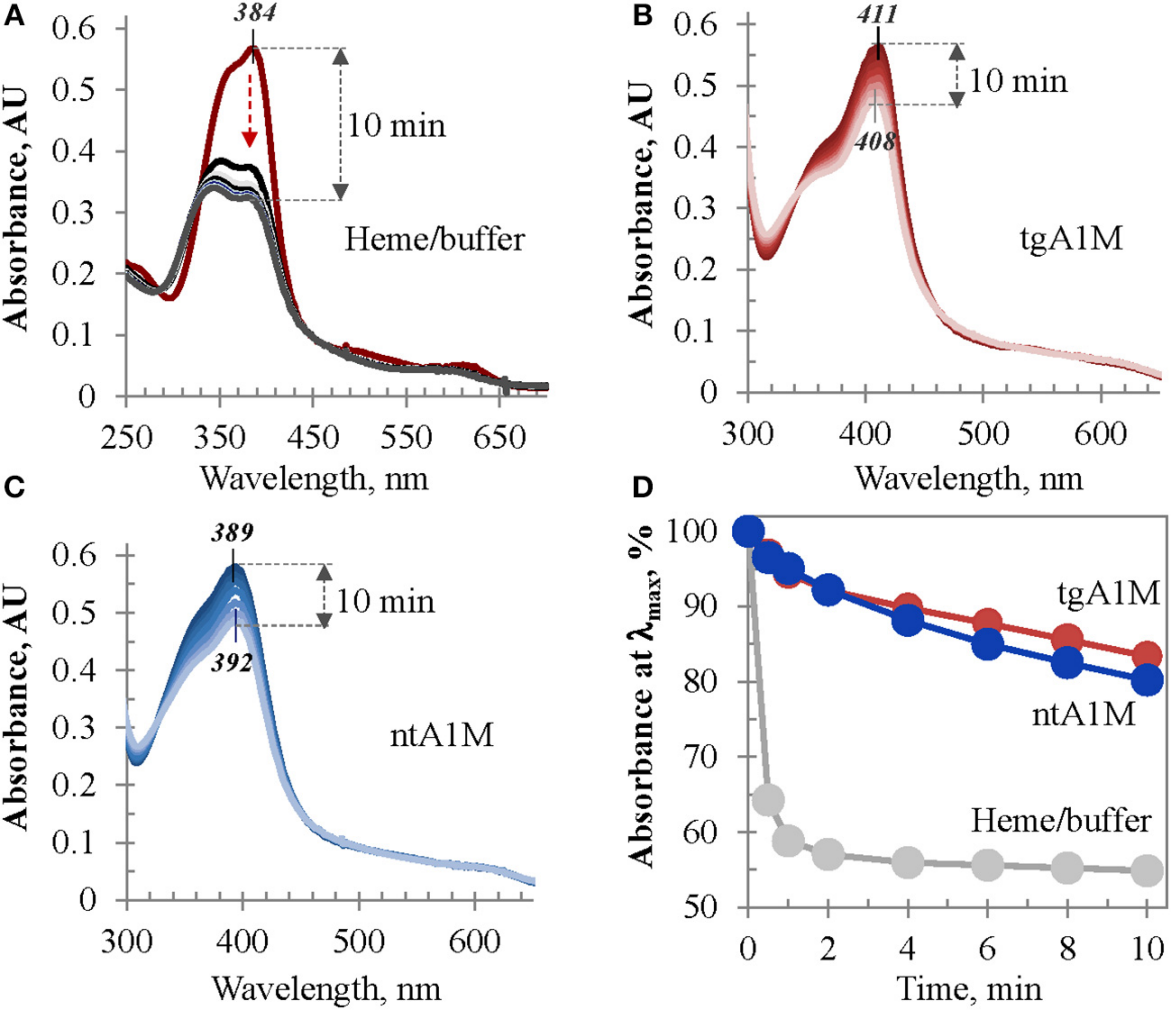
**Heme catalase-like activity. (A–C)** UV/Vis monitoring of the Soret band intensity of heme samples in Tris buffer alone **(A)**, and in complexes with tgA1M **(B)** and ntA1M **(C)**; **(D)** alterations of the Soret band initial intensity (%) measured over 10 min after addition of hydrogen peroxide. Heme complexes with tgA1M and ntA1M were prepared 20 h prior to these measurements, whereas heme/buffer sample of the same absorbance intensity was freshly prepared; for more details see Materials and Methods.

The heme/tgA1M and heme/ntA1M samples were assessed for catalase-like activity in order to evaluate the accessibility of heme in these complexes to hydrogen peroxide in comparison to that of free heme in buffer. As shown in Figure 8A, when an aliquot of hydrogen peroxide is added to a freshly prepared heme solution in Tris buffer, the intensity of its Soret band drastically drops within first 30 s, and 2 min later more than 42% of the initial intensity is lost. However, when the same amount of hydrogen peroxide is added to heme/tgA1M and heme/ntA1M solutions in Tris buffer, a very limited decrease of the Soret band intensity is observed for both samples (Figures 8B,C). Figure 8D provides a comparative summary for this functional assessment, suggesting that heme accessibility in the complexes with tgA1M and ntA1M is almost the same, and both A1M variants shield ferriheme from oxidation and partial decomposition by peroxide in a comparable fashion.

### Biological implications

Free heme exerts toxic effects in biological tissues mainly through oxidative reactions of the iron (Kumar and Bandyopadhyay, 2005). Heme causes tissue injury by catalyzing oxidation of proteins, lipids and DNA. Since the heme group is a lipophilic molecule it intercalates lipid bilayer membranes and destabilizes plasma membranes, mitochondria and nucleus. The primary targets of free heme in blood are the endothelial cells and kidneys. Thus, *in vivo* pro-inflammatory effects and renal toxicity have been reported in animals (Wagener et al., 2001; Rodriguez et al., 2003). The free heme toxicity in blood is probably mitigated to a large part by albumin and hemopexin, but A1M has recently been suggested among the innate defense molecules as having a role primarily in the extravascular compartments (Åkerström and Gram, 2014). Besides the heme-binding, A1M also yields antioxidative protection by radical-scavenging and reductase activity. Together, these three mechanisms gives the protein a powerful therapeutic potential and it was indeed shown that recombinant His-tagged A1M could be employed for *in vivo* treatment of the pregnancy disease preeclampsia, characterized by hypertension and kidney dysfunction, in a sheep model (Wester-Rosenlöf et al., 2014) and Hb-induced glomerular dysfunction in rats (Sverrisson et al., 2014).

## Conclusions

We have investigated heme interactions with recombinant A1M using UV/Vis absorption spectrometry, visible CD, SPR, migration-shift PAGE, and catalase-like activity assay. The latter four methods have not previously been applied for this purpose and provide a deeper insight into the dynamics and character of heme interactions with A1M. The results support the previously suggested two binding site model with binding affinities in the micromolar range. For the first time, induced visible CD visualizes the differences in the heme microenvironment at the A1M high and low affinity binding sites. The results also show that His-tagged and tagless A1M bind heme in a similar fashion, although the His-tag seems to be involved in the coordination with heme iron. This study thus provides an analytical platform for rapid evaluation of the ligand-binding properties of recombinant A1M versions including induced visible CD and an efficient SPR approach based on the capturing of tagged A1M by using anti-His-tag antibody, which also may be suitable for other recombinant proteins.

### Conflict of interest statement

The authors declare that the research was conducted in the absence of any commercial or financial relationships that could be construed as a potential conflict of interest.

## Abbreviations

A1M: α_1_-microglobulin
CD: circular dichroism
CE: Cotton Effect
DMSO: dimethyl sulfoxide
HA: human albumin
His8: histidine octapeptide
HPX: hemopexin
L/P: ligand-to-protein (molar) ratio
ntA1M: non-tagged alpha-1-microglobulin
PBS: phosphate buffer saline
SPR: surface plasmon resonance
tgA1M: His-tagged alpha-1-microglobulin
RU: response units.
